# Comorbidities and determinants of health on heart failure guideline-directed medical therapy adherence: All of us

**DOI:** 10.1016/j.ijcrp.2024.200351

**Published:** 2024-11-02

**Authors:** Trinh Do, Kyrillos Grace, Dawn Lombardo, Nathan D. Wong, Andy Y. Lee

**Affiliations:** a Division of Cardiology, Department of Medicine, School of Medicine, University of California, Irvine, CA, USA; b Department of Epidemiology and Biostatistics, University of California, CA, USA

**Keywords:** Heart failure, Guideline-directed medical therapy, HFrEF, HFpEF, Social determinants of health, All of us program

## Abstract

**Background:**

Heart failure with reduced ejection fraction (HFrEF) and heart failure with preserved ejection fraction (HFpEF) are challenging conditions to treat due to complex pathophysiology and associated comorbidities. However, recent trials have demonstrated improved outcomes with guideline-directed medical therapy (GDMT) for each subtype of heart failure.

**Objective:**

We investigated the relationship of determinants of health and risk factors with GDMT use for HFrEF and HFpEF in a large, diverse US cohort.

**Methods:**

Using the NIH-sponsored All of Us Program, we compared demographics, risk factors (e.g., hypertension, diabetes, smoking), and SDOH measures between HFrEF and HFpEF in US adults aged 18 years and older. We examined the proportions of HFrEF patients receiving fewer than four or all four GDMTs. HFpEF patients receiving two medications were compared with those receiving less than two recommended medications. Multiple logistic regression was used for data analysis.

**Result:**

Of 6049 HFrEF patients, 5838 (97 %) received fewer than four GDMTs, and 210 (3 %) received quadruple therapy. Of 3774 HFpEF patients, 162 (4 %) were on 2/3 GDMT, and only 38 (1 %) were on all three recommended medications. Patients with ASCVD and diabetes had higher odds of being on more than half of the recommended GDMT for both HFrEF and HFpEF. Additionally, females had higher odds of being on 2/3 GDMT for HFpEF (1.46 [1.08, 2.00]). Race, income, education, and health insurance types did not predict GDMT optimization.

**Conclusion:**

HFrEF and HFpEF GDMT remain underutilized. Future efforts to address comorbidities and system-wide healthcare interventions may improve heart failure GDMT.

## Abbreviations:

SDOH: social determinants of health
GDMT: guideline-directed medical therapy
HFrEF: heart failure with reduced ejection fraction
HFpEF: heart failure with preserved ejection fraction
ASCVD: atherosclerotic cardiovascular disease
ACEi: angiotensin-converting enzyme inhibitor
ARB: angiotensin II receptor blocker
ARNI: angiotensin receptor – neprilysin inhibitor
SGLT2i: sodium-glucose cotransport-2 inhibitor
MRA: mineralocorticoid receptor antagonist
NIH: national institutes of health
HF: heart failure
CKD: chronic kidney disease
EHR: electronic health records
OR: odd ratios
BMI: body mass index
ARIC: atherosclerosis risk in communities
T2DM: type 2 diabetes mellitus
LVEF: left ventricular ejection fraction
BNP: B-type natriuretic peptide
EF: ejection fraction
HFmrEF: heart failure with mildly reduced ejection fraction

## 1 Introduction

HF poses a significant burden on global healthcare systems, affecting millions of individuals [1]. The current types of HF referenced in major guidelines include HFpEF and HFrEF, along with GDMT, including ACEIs/ARBs/ARNI, beta-blockers, SGLT2i, and MRAs [2].

While promising results have been demonstrated for patients with HFrEF when GDMT is adequately followed, patients are often not being treated with appropriate prescriptions and doses in real-world data [[2], [3], [4], [5]]. The underutilization of GDMT in HFrEF and HFpEF has been recorded in multiple studies. The CHAMP-HF study highlighted that 27 % of eligible patients were not prescribed ACEI/ARB/ARNI, 33 % were not prescribed beta-blockers, and 67 % were not prescribed MRA therapy. Notably, only 13 % received ARNI, indicating significant underuse of ARNI and MRA therapy [5]. While social and economic characteristics were not independently associated with medication use or dose in the CHAMP-HF registry, the GWTG-HF registry highlighted the significant impact of social inequalities on health outcomes [6].

Our research aims to investigate the relationship between race, sex, comorbidities, and SDOH with GDMT adherence in patients with HFrEF or HFpEF using the NIH-funded All of Us database. One unique characteristic of All of Us is its extensive and inclusive health databases that focus on individuals from marginalized populations. It is anticipated that this database's inclusivity will allow us to observe how SDOH, such as lower income levels, lack of insurance, and lower education levels, are associated with poorer GDMT optimization. Additionally, it is hypothesized that patients with more comorbidities will be better optimized on GDMT than those without similar conditions.

## 2 Methods

### 2.1 The All of Us Research Program

The All of Us Research Program, initiated in May 2018, is a NIH-directed endeavor to establish a comprehensive and diverse database comprising a minimum of 1 million individuals in the United States. The primary objective of this initiative is to enhance the effectiveness of biomedical research and promote advancements in healthcare. Since its inception in May 2018, the program database has experienced substantial growth and currently encompasses more than 805,000 participants as of February 15, 2023. The database additionally emphasizes the comprehensive incorporation of health data on marginalized populations. Within the present cohort, it is noteworthy that a majority exceeding 75 % comprises individuals belonging to underrepresented demographics, with no less than 45 % representing racial minorities [7]. Participants were recruited and provided their EHR data and completed surveys on demographic and health-related information [7]. Additionally, informed consent was obtained from all participants, indicating their voluntary agreement to participate in the study. The present study employed de-identified data approved for utilization by researchers at the participating sites. A shared cloud-based environment enables researchers to readily access the data, fostering widespread data availability and facilitating dynamic data exploration and hypothesis testing [7].

### 2.2 Study population

The study population consisted of 9823 individuals aged 18 years and older, diagnosed with either chronic HFrEF or HFpEF by ICD-9/ICD-10 code, as obtained from the database. Participants with diagnoses that do not differentiate between diastolic or systolic HF and those with diagnoses falling under acute HF were excluded from the study. As the All of Us database has limited and incomplete records of BNP levels for all patients, excluding participants with unclear diagnoses can enhance the accuracy of categorizing participants into the correct grouping. We also chose to include only participants with chronic diagnoses and exclude those with acute diagnoses to avoid confounding variables such as contraindications of certain GDMT medications during acute exacerbation. However, this exclusion criterion inevitably reduced our sample size, which could potentially impact the significance of the results. Similarly, we excluded any participants with missing data or with answers that did not meet our coded condition (“Skip”, “I prefer not to answer”, “No matching concept”, “None of these” for race/ethnicity/gender). Patients with HFrEF were identified by ICD-9/ICD-10 codes for systolic HF (428.2/I50.2), chronic systolic HF (428.22/I50.22), chronic combined systolic and diastolic HF (428.42/I50.42), and HF with reduced ejection fraction. Patients with HFpEF were identified by ICD-10 codes for diastolic HF (428.3/I50.3), chronic diastolic HF (428.32/I50.32), and HF with a normal ejection fraction. The All of Us Research Program was approved by the institutional review boards of all participating sites and informed consent was provided by all participants. The current analysis utilized de-identified data approved for use by researchers at participating sites. *Our project involved the use of de-identified data so does not meet the definition of human subject research, therefore is exempt from IRB review.*

### 2.3 Medication

Full GDMT for HFrEF includes quadruple therapy with either an ACEi/ARB/ARNI, a beta-blocker, a SGLT2i, and an MRA. Current HFpEF therapy includes SGLT2i, ARNI, and MRA. We categorized participants with HFrEF into two groups: those prescribed 4/4 GDMT for HFrEF and those prescribed <4 GDMT for HFrEF (this includes individuals who are prescribed 3/4 GDMT for HFrEF, 2/4 GDMT for HFrEF, 1/4 GDMT for HFrEF, or 0/4 GDMT for HFrEF). Participants with HFpEF were also split into two groups: prescribed at least two GDMT for HFpEF (individuals taking 2/3 and 3/3 GDMT for HFpEF) and prescribed <2 GDMT for HFpEF (on 1/3 GDMT for HFpEF or 0/3 GDMT for HFpEF). We collected laboratory data, other comorbidities (diabetes, CKD, etc.) and/or risk factors (hypertension, dyslipidemia, etc.), and drug exposure information from EHR. Additionally, survey questions were used to gather demographic details and SDOH measures such as education level, income, insurance coverage, and smoking habits.

### 2.4 Statistical analysis

The R statistical package was employed within the research workbench for the All of Us Research Program. To examine the disparities in characteristics between the groups with HFrEF and HFpEF, the authors employed summary statistics and conducted an analysis of variance (ANOVA) and Chi-square tests of proportions to compare continuous and categorical variables across categories of GDMT, respectively. The Chi-squared test of proportions was used to compare demographic factors including risk groups, sex, race/ethnicity, health insurance, education, income categories, and smoking status across categories of GMDT use. Multiple logistic regression analyses were used to investigate the independent associations of demographic measures, risk factors, comorbidities, and social determinants of health with the odds of being on all 4 recommended therapies for HFrEF and any 2 of 3 recommended therapies for HFpEF. ORs and corresponding 95 % confidence intervals were computed to quantify the strength of these associations. Data analysis ensured that no groups or statistics had fewer than 20 participants, in accordance with the All of Us program researcher policy.

## 3 Results

Our analysis includes 9823 participants diagnosed with either HFrEF or HFpEF based on the inclusion criteria. Overall, 38.4 % had the diagnosis of HFpEF and 61.6 % with HFrEF. Our sample comprised 27.4 % non-Hispanic Black, 14.0 % Hispanic or Latino, 52.3 % non-Hispanic White, 1.1 % Asian participants, as well as 51.6 % males and 48.4 % females. In our sample, 2.9 % did not have health insurance and 24.4 % had Medicare. A total of 4976 (69.5 %) patients were from families with a household income of less than $50k. The mean age of our population was 67.1 (±12.6) years (Table 1). Most participants from this sample were obese (with a mean BMI of 33.0 kg/m2), diabetic (58.3 % with diabetes-related symptoms or taking diabetes medication), and with ASCVD history (71.4 %) (Table 2). Demographic characteristics were statistically different between the two HF groups. Participants who were diagnosed with HFpEF were more likely to be older (69.3 vs. 65.8 years, p < 0.001), female (59.3 % vs. 41.6 %, p < 0.001), non-Hispanic White (57.3 % vs. 49.1 %, p < 0.001) than those with HFrEF.

**Table 1:** Demographic characteristics of Patients with Heart Failure with Preserved Ejection Fraction and Reduced Ejection Fraction.

	HFpEF (N = 3774)	HFrEF (N = 6049)	Total (N = 9823)	p value
**Age, year**	69.3 (11.9)	65.8 (12.9)	67.1 (12.6)	<0.001
**Age group**				<0.001
18-<45 years	132 (3.5 %)	428 (7.1 %)	560 (5.7 %)	
45-<55 years	293 (7.8 %)	638 (10.5 %)	931 (9.5 %)	
55-<65 years	767 (20.3 %)	1490 (24.6 %)	2257 (23.0 %)	
65-<75 years	1209 (32.0 %)	1849 (30.6 %)	3058 (31.1 %)	
≥75 years	1373 (36.4 %)	1644 (27.2 %)	3017 (30.7 %)	
**Sex (male)**	1506 (40.7 %)	3460 (58.4 %)	4966 (51.6 %)	<0.001
**Race group**				<0.001
Other	195 (5.2 %)	325 (5.4 %)	520 (5.3 %)	
Non-Hispanic White	2161 (57.3 %)	2972 (49.1 %)	5133 (52.3 %)	
Non-Hispanic Black	876 (23.2 %)	1811 (29.9 %)	2687 (27.4 %)	
Hispanic or Latino	503 (13.3 %)	871 (14.4 %)	1374 (14.0 %)	
Asian	39 (1.0 %)	70 (1.2 %)	109 (1.1 %)	
**Education**				<0.001
Less than a high school degree or equivalent	443 (12.1 %)	845 (14.4 %)	1288 (13.5 %)	
Twelve Or GED	832 (22.7 %)	1548 (26.4 %)	2380 (25.0 %)	
College One to Three	1135 (31.0 %)	1795 (30.6 %)	2930 (30.7 %)	
College graduate or advanced degree	1257 (34.3 %)	1680 (28.6 %)	2937 (30.8 %)	
**Employment status**				0.138
Not currently employed for wages	680 (18.4 %)	1157 (19.6 %)	1837 (19.2 %)	
Employed for wages or self-employed	3016 (81.6 %)	4739 (80.4 %)	7755 (80.8 %)	
**Income**				0.002
less than 50k	1872 (67.1 %)	3104 (71.1 %)	4976 (69.5 %)	
50k∼100k	582 (20.9 %)	793 (18.2 %)	1375 (19.2 %)	
More than 100k	335 (12.0 %)	469 (10.7 %)	804 (11.2 %)	
**Health insurance**				<0.001
No	74 (2.0 %)	204 (3.5 %)	278 (2.9 %)	
Yes	3622 (98.0 %)	5680 (96.5 %)	9302 (97.1 %)	
**Health Insurance Type**				<0.001
Employer/Union/Commercial/Other	1282 (40.9 %)	1967 (39.9 %)	3249 (40.3 %)	
Medicaid	1007 (32.1 %)	1841 (37.3 %)	2848 (35.3 %)	
Medicare	844 (26.9 %)	1123 (22.8 %)	1967 (24.4 %)

**Table 2:** Comorbidities of patients with heart failure with preserved ejection fraction and reduced ejection fraction.

	HFpEF (N = 3774)	HFrEF (N = 6049)	Total (N = 9823)	p value
**ASCVD status**	2496 (66.2 %)	4513 (74.6 %)	7014 (71.4 %)	<0.001
**DM status**	2183 (57.9 %)	3543 (58.6 %)	5726 (58.3 %)	0.48
**Systolic blood pressure, mmHg**	132.0 (16.7)	127.3 (16.8)	129.1 (16.9)	<0.001
**Diastolic blood pressure, mmHg**	75.0 (10.2)	75.8 (10.6)	75.5 (10.5)	<0.001
**HTN**	1990 (52.7 %)	2735 (45.2 %)	4725 (48.1 %)	<0.001
**Left ventricular ejection fraction, %**	58.7 (12.2)	43.1 (15.6)	49.2 (16.2)	<0.001
**BNP, pg/mL**	245.9 (241.0)	319.1 (264.2)	288.5 (257.3)	<0.001
**BMI, kg/m2**	34.0 (9.8)	32.4 (14.0)	33.0 (12.6)	<0.001
**Glucose, mg/dL**	132.6 (42.9)	133.7 (44.4)	133.3 (43.8)	0.247
**Hemoglobin A1c, mg/dL**	6.7 (2.0)	6.7 (1.7)	6.7 (1.8)	0.133
**Smoking**				<0.001
Non-smoker	1774 (48.8 %)	2606 (44.8 %)	4380 (46.3 %)	
Former smoker	1393 (38.3 %)	2164 (37.2 %)	3557 (37.6 %)	
Current smoker	469 (12.9 %)	1047 (18.0 %)	1516 (16.0 %)	
**LDL-C category**				<0.001
<70 mg/dL	616 (22.2 %)	1322 (28.2 %)	1938 (26.0 %)	
70-<100 mg/dL	1089 (39.2 %)	1785 (38.1 %)	2874 (38.5 %)	
≥100 mg/dL	1073 (38.6 %)	1582 (33.7 %)	2655 (35.6 %)	

HFrEF – Heart failure with reduced ejection fraction; HFpEF – heart failure with preserved ejection fraction; ASCVD - atherosclerotic cardiovascular disease; DM – Diabetes; HTN – hypertension; BNP – brain natriuretic peptide; BMI – body mass index; LDL-C – low density lipoprotein C.

**Fig. 1:**
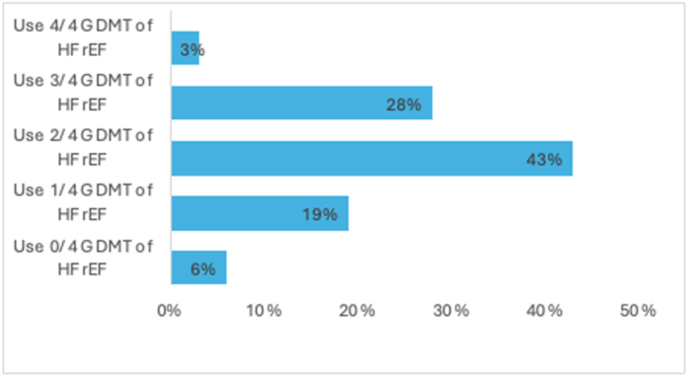
Percentage of Participants on four groups of GDMT for HFrEF. **Caption:** Total participants with HFrEF: 6049. Of the total participants with HFrEF, 6 % are on 0 out of the 4 GDMT, 19 % are on 1 out of the 4 GDMT, 43 % are on 2 out of the 4 GDMT, 28 % are on 3 out of the 4 GDMT, and 3 % are on the quadruple therapy (all 4 GDMT). GDMT = guideline-directed medical therapy; HFrEF = heart failure with reduced ejection fraction.

From our sample, 6049 patients were identified with HFrEF. Of those patients, 5838 (97 %) patients were on less than 4 GDMT, while 210 (3 %) patients were on quadruple therapy (Fig. 1). Multiple logistic regression showed participants with ASCVD, and diabetes had higher odds (95 % CI) of being on quadruple therapy, (OR = 2.19 [1.28, 3.99]) and (OR = 9.07 [4.88, 18.84]), respectively, while hypertension had lower odds, (OR = 0.64 [0.43, 0.95]). Race, income, education, and health insurance types did not predict medical therapy optimization (Table 3).

**Table 3:** Multiple logistics regression of being on 4/4 GDMT for HFrEF.

Variables	Use 4/4 of GDMT for HFrEF odds ratio [95 % CI]
Age (per year)	0.97 [0.95, 0.98]
Female sex	0.94 [0.63, 1.40]
Race/Ethnicity (Asian/other)	0.67 [0.25, 1.50]
Race/Ethnicity (Non-Hispanic Black)	0.92 [0.55, 1.53]
Race/Ethnicity (Hispanic or Latino)	1.19 [0.64, 2.16]
ASCVD	2.19 [1.28, 3.99]
DM	9.07 [4.88, 18.84]
HTN	0.64 [0.43, 0.95]
Smoking history	0.60 [0.30, 1.10]
BMI (per kg/m2)	1.01 [0.99, 1.01]
Income (50k∼100k)	1.72 [1.00, 2.92]
Income (More than 100k)	1.74 [0.88, 3.36]
Education (Twelve Or GED)	0.76 [0.37, 1.63]
Education (College One to Three)	1.13 [0.58, 2.33]
Education (College graduate or advanced degree)	1.11 [0.53, 2.40]
Health insurance (Medicaid)	0.92 [0.54, 1.57]
Health insurance (Medicare)	0.74 [0.41, 1.28]

Reference groups**:** Gender—female, race—non-Hispanic White, ASCVD—no ASCVD, DM—no DM, HTN—no HTN, smoking history—no smoking history, income—<50 k, education—less than a high school degree or equivalent, health insurance—employer/purchased.

HFrEF – Heart failure with reduced ejection fraction; HFpEF – heart failure with preserved ejection fraction; ASCVD - atherosclerotic cardiovascular disease; HTN – hypertension; BMI – body mass index; DM - diabetes.

**Fig. 2:**
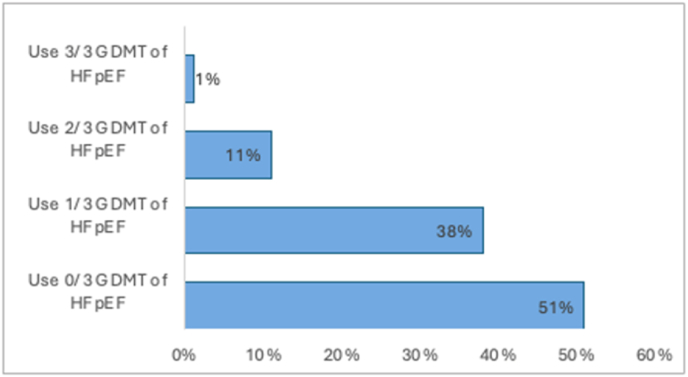
Percentage of Participants of three groups of GDMT for HFpEF. **Caption**: Total participants with HFpEF: 3774. Of the total participants with HFpEF, 51 % are on 0 out of the 3 recommended GDMT, 38 % are on 1 out of 3 recommended GDMT, 11 % are on 2 out of the 3 recommended GDMT, 1 % are on all three recommended medications. GDMT = guideline-directed medical therapy; HFpEF = heart failure with preserved ejection fraction.

In the HFpEF group, 3736 (99 %) participants were on less than 3 GDMT agents, with 162 (4 %) being on 2/3 GDMT, and only 38 (1 %) individuals on all three recommended medications (Fig. 2). Multiple logistic regression analysis revealed greater odds of being on at least 2 GDMT for HFpEF among participants with known ASCVD, (OR = 1.96 [1.41, 2.78]), diabetes, (OR = 2.15 [1.56, 3.01]), and BMI ≥35 kg/m^2^ (OR = 1.02 [1.01, 1.04]). In addition, female sex was also associated with increased odds of being on at least two agents (OR = 1.46 [1.08, 2.00]). The likelihood of being prescribed GDMT for HFpEF was not predicted by race, education, income, or health insurance type (Table 4).

**Table 4:** Multiple Logistics Regression of Being on minimum of 2/3 GDMT for HFpEF.

Variables	Use a minimum of 2/3 of GDMT for HFpEF odds ratio [95 % CI]
Age (per year)	1.01 [1.00, 1.02]
Female sex	1.46 [1.08, 2.00]
Race/Ethnicity (Asian/other)	1.21 [0.59, 2.29]
Race/Ethnicity (Non-Hispanic Black)	1.39 [0.98, 1.98]
Race/Ethnicity (Hispanic or Latino)	1.47 [0.87, 2.42]
ASCVD	1.96 [1.41, 2.78]
DM	2.15 [1.56, 3.01]
HTN	1.24 [0.94, 1.65]
Smoking History	0.76 [0.47, 1.20]
BMI (per kg/m2)	1.02 [1.01, 1.04]
Income (50k∼100k)	1.09 [0.72, 1.63]
Income (More than 100k)	1.21 [0.71, 2.00]
Education (Twelve Or GED)	1.13 [0.66, 2.00]
Education (College One to Three)	1.27 [0.75, 2.22]
Education (College graduate or advanced degree)	1.00 [0.57, 1.81]
Health insurance (Medicaid)	1.19 [0.80, 1.78]
Health insurance (Medicare)	0.79 [0.54, 1.14]

Reference groups: Gender—female, race—non-Hispanic White, ASCVD—no ASCVD, DM—no DM, HTN—no HTN, smoking history—no smoking history, income—<50 k, education—less than a high school degree or equivalent, health insurance—employer/purchased.

HFrEF – Heart failure with reduced ejection fraction; HFpEF – heart failure with preserved ejection fraction; ASCVD - atherosclerotic cardiovascular disease; HTN – hypertension; BMI – body mass index; DM - diabetes.

## 4 Discussion

Our study in a large real-world cohort of US adults of diverse backgrounds shows that across HF categories, sex, and ethnic groups, there remains significant underutilization of GDMT for HFrEF and HFpEF, with only 3 % of participants with HFrEF are currently on the quadruple therapy and 1 % of participants with HFpEF are on 3/3 recommended GDMT. SGLT2i and ARNI are shown to be the least utilized. Our study shows various comorbidities as well as female sex are associated with a greater likelihood of being on GDMT.

According to our results, only a small percentage of the population reports to be on 4/4 GDMT for HFrEF, with many participants on 2/4 medications. This finding is similar to the ARIC study, in which the prevalence of optimal and acceptable GDMT for HFrEF was 5.5 % and 54.4 %, respectively, but did not differ significantly by socioeconomic status [8]. Evidence also suggests that a significant number of patients with HFrEF are started on GDMT at the time of hospital discharge or in the outpatient setting, and most eligible HFrEF patients are not prescribed target-dose GDMT.^9,10^Among the four GDMTs, our results are consistent with other studies that demonstrated that SGLT2i and ARNI are the two least utilized [10]. The findings of the Evolution HF study indicated that the mean time to initiate the newer GDMTs, namely dapagliflozin (SGLT2i) or sacubitril/valsartan (ARNI) after HF diagnosis or hospitalization for HF tends to be longer compared to other established GDMTs [11]. Despite the findings of our study suggesting that the utilization of SGLT2i is comparatively lower, it is noteworthy that patients with comorbidities such as ASCVD and T2DM exhibit a higher probability of being on quadruple therapy for HFrEF. Numerous research studies have substantiated the advantageous application of these newer agents in individuals with T2DM and HFrEF [[12], [13], [14]]. The benefits were observed in individuals diagnosed with HFrEF, regardless of whether they had T2DM, as evidenced by the findings of the DAPA-HF study [13]. Furthermore, hospitalizations and death rates from cardiovascular causes were significantly reduced with Sotagliflozin therapy in the SOLOIST-WHF trial [15]. However, given that SGLT2i entered clinical guidelines only recently after its efficacy was elucidated in both randomized clinical trials DAPA-HF in 2020 and EMPEROR-Reduced in 2021, the results from our study are not surprising [[16], [17], [18]]. Changes in treatment strategies are expected to improve over time.

In the context of HFpEF, a similar trend to that of HFrEF was observed in which the majority of participants were on less than 3 GDMT and only 1 % of the study population taking 3/3 of GDMT. Similar to how SGLT2i was shown to be beneficial in HFrEF mortality reduction, SGLT2i use also improves health status and quality of life in HFpEF in recent clinical trials, such as DELIVER and EMPEROR-Preserved [19,20]. The results of this study highlight the potential significance of SGLT2i in the management of HFpEF, suggesting that they may have additional advantages compared to MRAs and ARNIs [21,22]. The effectiveness of ARNI treatment in patients with HFpEF was evaluated in the PARAGON-HF trial [23]. While the ARNI treatment did not yield a statistically significant reduction in hospitalization for HF and cardiovascular mortality, it is worth noting that patients who had recently been hospitalized experienced greater benefits from ARNI treatment. Additionally, a more pronounced benefit was observed in female patients compared to their male counterparts [12]. This observation could potentially explain why female sex is associated with being on more than half of GDMT in our study.

Our results reveal that GDMT prescriptions for both HFrEF and HFpEF are seen to be higher in those with comorbidities such as T2DM, ASCVD, and obesity compared to those without multiple risk factors. Current literature shows that there is a bidirectional relationship between HF comorbidities whereby the presence of one increases the severity of the other [13], and the prognosis is worse when both are present. Interestingly, a study on a multimorbid patient population (West Tokyo HF Registry: WET‐HF) revealed that the benefit of GDMT was not affected by comorbidities, but the association weakened as the comorbidity burden increased [24]. This could potentially explain the under-optimization of GDMT in patients with more than one comorbidity. We did not analyze the burden of comorbidities for each participant and correlate it with the number of GDMT medications they were taking. For example, existing literature has shown the challenge in ACEi/ARB up-titrating in patients with CKD, which could lead to poorer safety and more side effects [25]. Similar findings are demonstrated in the EPIC-HF trial.

Unlike current trends in the literature, our findings show that age, sex, race, and various social determinants of health, such as income, education level, and insurance status, do not predict whether an individual with HFrEF will be prescribed optimized GDMT. An intriguing observation was made, as we had hypothesized a more robust inverse relationship between these SDOHs and the number of medications, particularly in the All of Us database due to the inclusion of marginalized populations. Existing literature shows that low-income patients and those residing in lower socioeconomic status neighborhoods with HF exhibited an approximately twofold increase in the likelihood of in-hospital mortality and post-discharge events compared to the high-income group, which can be partially attributed to lower GDMT utilization [[26], [27], [28]]. Furthermore, educational attainment demonstrated a significant association with GDMT usage at discharge and during follow-up [29]. One explanation for our study's insignificant findings between SDOH and medication optimization may be attributed to the population characteristics, where a substantial proportion (69.5 %) of participants with HF have an income below $50k. Additionally, participation in the All of Us program is voluntary, and those who consent to be in the database may already have better access to healthcare, irrespective of their race, income, education level, and insurance type. While SDOHs are crucial determinants of access to healthcare services, they do not always directly translate into healthcare utilization. Even with access to healthcare, factors such as healthcare literacy, patient-provider communication, and personal beliefs about treatment can significantly impact utilization.

In a prior investigation, researchers noted that female sex was consistently linked to inadequate utilization (defined as being on 0/4, 1/4, and 2/4 GDMT) [30]. In addition, a recent study confirms the sex disparities in longitudinal use and intensification of GDMTs in patients with newly diagnosed HFrEF with lower GDMT usage in female patients [31]. Our findings of the association between female sex and higher odds of being on more medications for HFpEF can be explained through the sex-specific differences in diagnosing HFpEF. In contrast to their male counterparts, women diagnosed with HFpEF exhibit a greater prevalence of pronounced dyspnea symptoms and a higher likelihood of experiencing a deteriorated health status [32]. The physical examination for individuals with HFpEF is typically comparable between both sexes. Nevertheless, there may be notable distinctions based on sex that can be identified through diagnostic testing. In the context of echocardiographic imaging, women tend to exhibit smaller left ventricular chamber size and consequently demonstrate higher LVEF compared to men because of increased concentric remodeling [[32], [33], [34]]. Greater years of survival lost to HF post-acute HF hospitalization in females compared to median age- and sex-matched US males also explains the higher probability for medication optimizations [35].

The underutilization of GDMTs in both HFrEF and HFpEF can be attributed to various factors. Nevertheless, significant endeavors have been made to optimize medications for patients. Interdisciplinary heart failure clinics have been established to specifically address patients who have subtherapeutic dosages of GDMTs or who initiate GDMT after being discharged from the hospital. These clinics have been proven to assist in increasing medication dosage to reach the desired level, leading to reduced rates of readmission for heart failure or death from any cause [36]. Additional interventions, such as educating clinicians and patients, did not consistently show improvements in GDMT. EHR alerts and audits enhanced specific aspects of GDMTs but lacked consistency across all components [9,37]. In our analysis, it has been revealed that ASCVD and T2DM could be significant factors associated with optimizing GDMT. Interestingly, our findings indicated that SDOH factors such as income level, education attainment, and insurance status did not predict HF management in the All of Us database. This unique discovery enables us to further explore an individual's physiological limitations and intolerance to GDMT. In essence, physicians should exercise caution when balancing the optimization of GDMT for HF and addressing contraindications.

Our study has several strengths and limitations. An important strength is the inclusion of participants who accurately reflect the heterogeneous demographics of the United States. This encompasses individuals who have historically faced exclusion or underrepresentation in the field of health research. The integration of medical records in a comprehensive manner allows for the assessment of medication optimization in individuals with both HFrEF and HFpEF, across different groups that are classified based on their comorbidities and SDOH. Given the scarcity of available data on BNP values and EF percentages, the determination of HFrEF and HFpEF was primarily reliant upon the diagnoses documented in the participant's medical records. The trial relies on ICD-10 codes to determine which category of HF the patient has been diagnosed without a corresponding EF to verify this, resulting in diagnostic uncertainty. As in this case, HFmrEF (EF of 40%–49 %) might have been added to the subgroup HFpEF. Furthermore, our database lacks comprehensive data on left ventricular ejection fraction and pro-BNP, which hinders the accurate differentiation between HFrEF and HFpEF. The participant categorization was solely based on their officially recorded diagnosis, which may not be entirely accurate. Furthermore, our cohort lacked sufficient data points for eGFR to accurately categorize each participant into a kidney function group. Impaired kidney function has been repeatedly identified as a significant barrier to GDMT optimization. In addition, we did not take into consideration other factors that limit GDMT such as azotemia, hyperkalemia, or hypotension. Consequently, the limited number of participants with optimized GDMT could be attributed to comorbidities that restrict these patients from receiving GDMT. Another limitation inherent in our study is its utilization of a cross-sectional design, which prevents the ability to incorporate multiple assessments for evaluating adherence or conducting follow-up investigations into cardiovascular or mortality outcomes, thus limiting the generalizability. There are also other limitations in using electronic health records (EHR) data, where there may be inconsistencies across study sites in capturing prescription and diagnostic data. Lastly, our study population primarily comprises underserved and disadvantaged individuals, which may yield different results compared to health claims data obtained from insured individuals.

## 5 Conclusions

In conclusion, we demonstrate continued underutilization of GDMT for both HFrEF and HFpEF within a diverse sample of the US population based on data from the All of Us Research Program, warranting the improving use and adherence of GMDT. The staggering underutilization of crucial medications, particularly SGLT2i and ARNI, reveals a substantial gap in optimizing HF treatment. Future studies should be directed toward personalized interventions aimed at addressing these barriers and ultimately standardizing our approach to prescribing GDMT such that all patients with HF are on the optimal regimen of medications barring any medical contraindications. Among these strategies should be patient education initiatives, comprehensive provider training programs, insurance coverage, and systemic changes at the healthcare level. Bridging the communication gap between healthcare providers and patients through innovative tools and patient-centered communication approaches will be paramount in ensuring a more widespread and effective adoption of GDMT.

## CRediT authorship contribution statement

**Trinh Do:** Writing – original draft, Software, Methodology, Investigation. **Kyrillos Grace:** Writing – original draft, Investigation. **Dawn Lombardo:** Writing – review & editing, Supervision, Conceptualization. **Nathan D. Wong:** Writing – review & editing, Software, Resources, Conceptualization. **Andy Y. Lee:** Writing – review & editing, Writing – original draft, Supervision, Methodology, Conceptualization.

## Informed consent statement

Informed consent was obtained from all subjects involved in the study.

## Disclosure

All the authors have no relationships with industry or any other financial disclosure.

## Institutional review board statement

Our project involved use of de-identified data so does not meet the definition for human subject's research, therefore is exempt from IRB review.

## Data availability

All data utilized in this study are available from the All of Us Research Program at https://allofus.nih.gov/(accessed on 15 February 2023).

## Funding

The *All of Us* Research Program is supported by the National Institutes of Health, Office of the Director: Regional Medical Centers: 1 OT2 OD026549; 1 OT2 OD026554; 1 OT2 OD026557; 1 OT2 OD026556; 1 OT2 OD026550; 1 OT2 OD 026552; 1 OT2 OD026553; 1 OT2 OD026548; 1 OT2 OD026551; 1 OT2 OD026555; IAA #: AOD 16037; Federally Qualified Health Centers: HHSN 263201600085U; Data and Research Center: 5 U2C OD023196; Biobank: 1 U24 OD023121; The Participant Center: U24 OD023176; Participant Technology Systems Center: 1 U24 OD023163; Communications and Engagement: 3 OT2 OD023205; 3 OT2 OD023206; and Community Partners: 1 OT2 OD025277; 3 OT2 OD025315; 1 OT2 OD025337; 1 OT2 OD025276.

## Declaration of competing interest

The authors of this manuscript do not have any conflicts of interest.
