# Supplementary material for: Comorbidities and determinants of health on heart failure guideline-directed medical therapy adherence: All of us
Source: Int J Cardiol Cardiovasc Risk Prev. 2024 Nov 2;23:200351. doi: 10.1016/j.ijcrp.2024.200351 (PMC11577182; doi:10.1016/j.ijcrp.2024.200351)
Supplement: Multimedia component 1 [file mmc1.docx]

**Supplemental Table and Figure:**

**Table 5: Medication Use in Patients with HFrEF and HFpEF**

|  | **HFpEF (N=3774)** | **HFrEF (N=6049)** | **Total (N=9823)** | **p value** |
| --- | --- | --- | --- | --- |
| **Beta Blocker Use** | 2482 (65.8%) | 4333 (71.6%) | 6815 (69.4%) | < 0.001 |
| **SGLT2i Use** | 177 (4.7%) | 429 (7.1%) | 606 (6.2%) | <0.001 |
| **ACEi/ARB/ARNI Use** | 2633 (69.8%) | 5077 (83.9%) | 7710 (78.5%) | < 0.001 |
| **MRA Use** | 802 (21.3%) | 2430 (40.2%) | 3232 (32.9%) | < 0.001 |

HFrEF – Heart failure with reduced ejection fraction; HFpEF – heart failure with preserved ejection fraction; SGLT2i – sodium-glucose cotransporter-2 inhibitor; ACEi – angiotensin-converting-enzyme inhibitor; ARB – angiotensin II receptor blocker; ARNI – angiotensin receptor neprilysin inhibitor; MRA – mineralocorticoid receptor antagonist.

**Table 6: HFpEF Logistic Regression Main Findings for 2/3 GDMT**


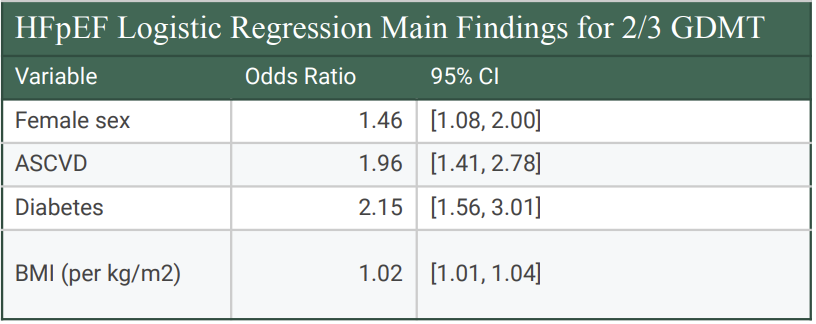


HFpEF – Heart failure with preserved ejection fraction; ASCVD - atherosclerotic cardiovascular disease; BMI – body mass index

**Table 7: Logistic Regression Main Findings for 4/4 GDMT**


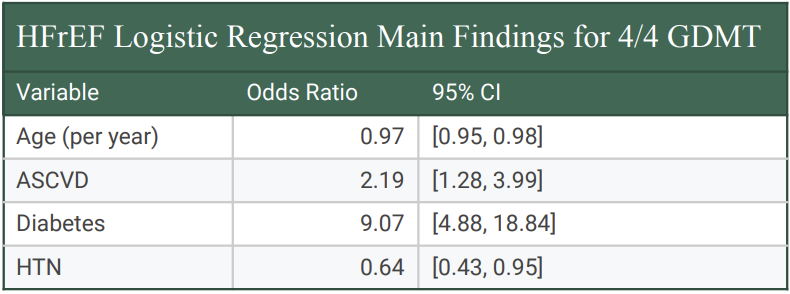


HFrEF – Heart failure with reduced ejection fraction; ASCVD - atherosclerotic cardiovascular disease; HTN – hypertension

**Figure 3: Flow chart of participant selection process**

**
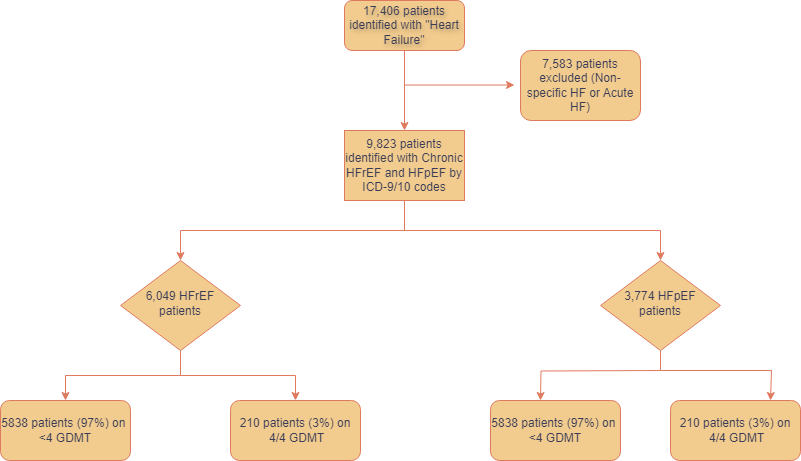
**

**Figure 4: Logistic Regression of Participants on 4/4 GDMT Use in HFrEF**

**
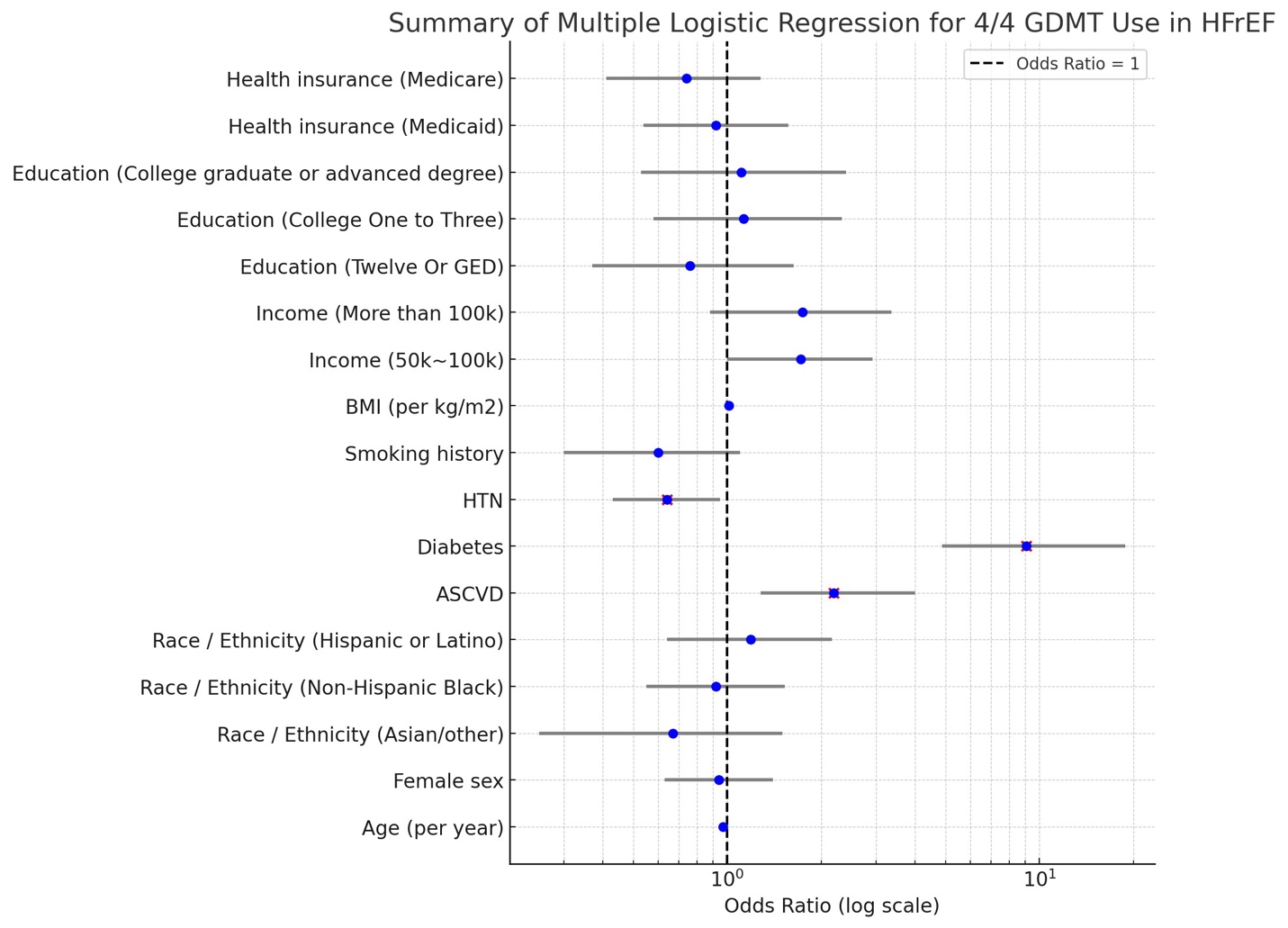
**

**Figure 5: Logistic Regression of Participants on 2/3 GDMT Use in HFpEF**

**
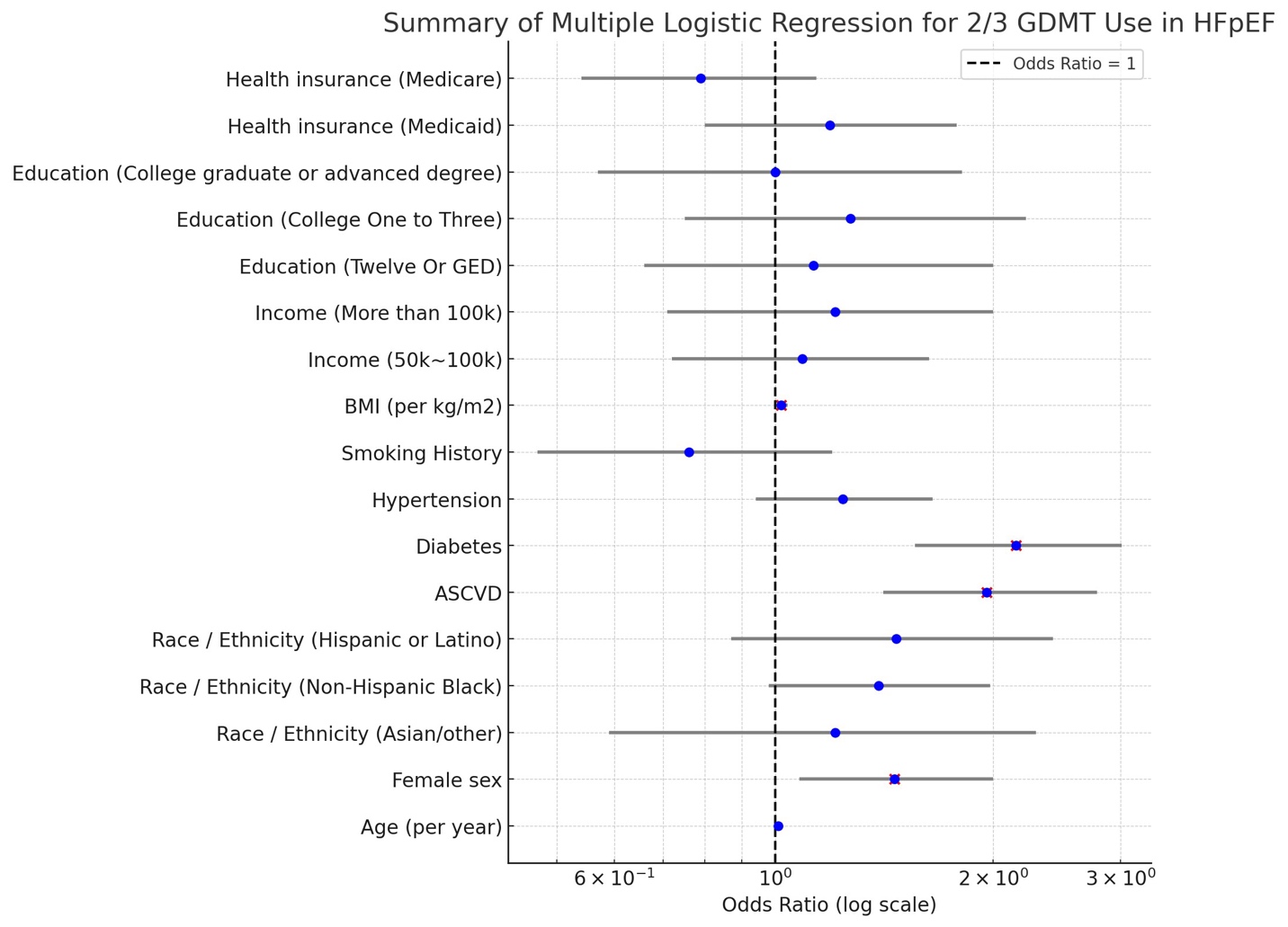
**
